# At a Crossroads: Opioid Use Disorder, the X-Waiver, and the Road Ahead

**DOI:** 10.31486/toj.23.0074

**Published:** 2024

**Authors:** Jaskaran Singh Dhillon, Leah Feulner, Ariya Beitollahi, Kelly Kossen, David Galarneau

**Affiliations:** ^1^ The University of Queensland Medical School, Ochsner Clinical School, New Orleans, LA; ^2^ Department of Psychiatry, Ochsner Clinic Foundation, New Orleans, LA

**Keywords:** *Addiction medicine*, *analgesics–opioid*, *buprenorphine–naloxone drug combination*, *opioid-related disorders*, *psychiatry*

## Abstract

**Background:** Buprenorphine/naloxone (Suboxone) is widely considered the first-line treatment for opioid use disorder (OUD), which causes significant morbidity and mortality in the United States, but prior to 2023, practitioners interested in prescribing buprenorphine/naloxone for OUD needed a special Drug Enforcement Administration certification (the X-Waiver) that imposed a patient cap and other limitations. The Consolidated Appropriations Act of 2023 considerably decreased the restrictions on prescribing practitioners. Buprenorphine/naloxone can now be prescribed like any other prescription opioid, excluding methadone. The historic context for the opioid crisis, OUD, the X-Waiver, and additional initiatives that may be needed beyond legislative change to effectively address OUD are the subjects of this review.

**Methods:** To develop this review of the opioid crisis, OUD, and OUD treatment, we conducted a literature search of the PubMed database and constructed a timeline of the opioid crisis and changes in OUD treatment, specifically the X-Waiver, to characterize the historic context of OUD and the X-Waiver against the background of the opioid crisis.

**Results:** The opioid crisis has had pervasive public health and economic impacts in the United States. Major changes to the treatment of OUD have occurred as a result of the Drug Addiction Treatment Act of 2000 that imposed the X-Waiver and the Consolidated Appropriations Act of 2023 that repealed the X-Waiver.

**Conclusion:** The repeal of the X-Waiver is predicted to increase the accessibility of buprenorphine/naloxone in the United States. However, additional work beyond legislative change, including institutional support and reduction of stigma and disparities, is needed to substantially improve outcomes for OUD patients.

## INTRODUCTION

Opioid use disorder (OUD) is a complex chronic disorder, characterized by patterns of remission, recurrence, compulsive use, and continued use despite associated harm. Left unchecked, OUD causes significant morbidity and mortality.^1^ OUD is also associated with legal, interpersonal, and employment problems.^2^ The increase in OUD in the United States is attributable to a variety of factors, such as widespread access to prescription opioid analgesics and illicit fentanyl and fentanyl analog use.^3^ In 2019, the number of adults in the United States with OUD was estimated to be between 6.7 million and 7.6 million.^4^ In 2020, approximately 75% of 91,799 drug overdose deaths involved an opioid, and the number of overdose deaths continued to rise during the coronavirus disease 2019 (COVID-19) pandemic, with an unprecedented 107,477 overdose deaths in the 12-month period ending in August 2022.^5,6^ The economic burden is considerable as well. In 2018, OUD-related costs in the United States were estimated to be $786.8 billion to society, $93 billion to taxpayers, and $89.1 billion to the health care sector.^7^ Statistics such as these led Xavier Becerra, 25th Secretary of the US Department of Health and Human Services, to renew, effective February 2023, a declaration proclaiming that an opioid “public health emergency exists and has existed since January 27, 2020, nationwide.”^8^

Among the medications that have been used to address OUD, buprenorphine/naloxone (Suboxone) has proven to be an efficacious combination drug; buprenorphine is an opioid partial agonist used for opioid replacement therapy, and the coformulation with the opioid antagonist naloxone may help prevent parenteral abuse.^9^ The Drug Addiction Treatment Act of 2000 (DATA 2000), passed by the 106th Congress on July 19, 2000, allowed physicians to prescribe buprenorphine but only under strict requirements that included mandatory structured education and acquisition of a prescribing identification number (ie, an X-Waiver, also called an X-number or X-license) from the Drug Enforcement Administration (DEA).^10^ As a result of these restrictions, buprenorphine prescribing remained low, and the OUD public health crisis continued. On December 29, 2022, with the signing of the Consolidated Appropriations Act of 2023, the DEA and the Substance Abuse and Mental Health Services Administration eliminated the X-Waiver, so that any provider with a standard DEA registration number could issue buprenorphine prescriptions. According to a letter from DEA Administrator Anne Milgram, the legislative repeal would “increase access to buprenorphine for those in need” by eliminating the X-Waiver and the caps on the number of patients a prescriber could treat for OUD with buprenorphine.^11^ However, the impact of the X-Waiver repeal remains to be determined.

## METHODS

This review provides background on opioids and OUD, including the historic and current course of opioid addiction trends and statistics. A primary focus is the repeal of the X-Waiver requirement and the possible implications for patients being treated for OUD. We also established a historic timeline of the opioid crisis and changes to the X-Waiver. For this review, we conducted a literature search of the PubMed database for peer-reviewed publications and government documents on the topics of “OUD,” “X-Waiver,” and related terms. Subtopics related to OUD, such as the cost of care burden, were excluded as these subtopics were outside of the scope of this review and best suited to future work.

## OPIOID USE AND MISUSE

### Opium, Opiates, and Opioids

Opium is derived from the seed capsule of the opium poppy, *Papaver somniferum.* Its discovery and use for both medicinal and recreational purposes can be traced to prehistoric times, as early as the Neolithic Age of the 4th millennium BC.^12^ Opium was commonly used as an anesthetic and for the treatment of ailments such as pain, diarrhea, and melancholy.^12^ In the early 1800s, morphine was isolated from opium, and in 1874, heroin was synthesized.^12^ Produced by the Bayer Company on a commercial scale in 1898, heroin, considered a “wonder drug” compared to codeine for the treatment of respiratory disease, soon became a drug of abuse as well.^13^ Synthetic agents such as oxycodone, hydromorphone, and hydrocodone were gradually introduced in the early 20th century, and fentanyl became available in the United States in 1968.^14^

A degree of clarification is necessary when discussing opioids, as terms such as opiates, opioids, narcotics, and pain pills are often used interchangeably in the literature. The term *opiates* refers to compounds extracted or refined directly from plant matter such as poppy sap and fibers: opium, morphine, codeine, and heroin. On the other hand, the term *opioids* commonly refers to compounds partially or entirely synthesized in laboratories: hydrocodone, oxycodone, oxymorphone, methadone, and fentanyl.^15^ However, the term *opioid* is also commonly used as a general term for any agent that binds to and activates opioid receptors.^16^ Fentanyl and its various synthesized analogs have also been classified as novel synthetic opioids or as new psychoactive substances.^17^

The pharmacologic action of opioids is well established in the scientific literature. Opioids exert their activity via various opioid receptors located in the central and peripheral nervous system: delta receptors (with analgesia, antidepressant, and physical dependence–related effects), kappa receptors (with analgesia, depression, diuresis, miosis, and sedation–related effects), and mu receptors (with analgesia, physical dependence, respiratory depression, miosis, euphoria, and reduced gastrointestinal motility–related effects).^18^ Of the 3 receptor classes, mu receptors are the most connected with addiction.

### Opioid Use Disorder

OUD involves the use of illicit agents such as heroin and fentanyl or of prescription medications such as oxycodone and hydrocodone that are prescribed to treat acute and chronic pain.^3^ Heroin is often injected but can be smoked or snorted, fentanyl is usually injected, and other opioids are taken via injection or mouth.^19^ Unknown to users, recreational drugs such as heroin may also be laced with fentanyl.^20^ Physical dependence is a key facet of OUD, and withdrawal from opioids is characterized by symptoms such as insomnia, diarrhea, nausea, vomiting, dysphoria, and anxiety.^21^ Chronic use may cause opioid-induced hyperalgesia, a state of nociceptive sensitization that has the paradoxical effect of causing increased sensitivity to painful stimuli.^22^ Of great concern, tolerance to the analgesic and hedonic effects of opioids develops faster than tolerance to respiratory depression.

The *Diagnostic and Statistical Manual of Mental Disorders, Fifth Edition, Text Revision* defines OUD as a concerning pattern of “use leading to clinically significant impairment or distress,” with diagnostic criteria including overuse; craving; social, occupational, or recreational problems; physical hazard; tolerance; and withdrawal.^19^ Acute opioid intoxication may present as problematic behavioral and psychological changes, with pupillary constriction, drowsiness or coma, slurred speech, impaired attention or memory, and possible perceptual disturbances.^19^ Individuals addicted to opioids experience mortality rates approximately 6 to 20 times greater than the general population, with overdose-caused respiratory depression the most common cause of death.^23^

Risk factors for developing OUD are similar to those for other substance use disorders, such as younger age, male sex, lower educational attainment, lower income level, unemployment, and comorbid psychiatric conditions such as anxiety or another substance use disorder.^24^ Social and environmental factors also contribute to an increased susceptibility to OUD, including exposure in utero; having parents with OUD; having a personality oriented toward novelty or deviance; initiation, experimentation, or escalation of drug use during adolescence; and influence from peer groups.^25^ Associations have been found between OUD and adverse childhood experiences, preadolescent sexual abuse, and posttraumatic stress disorder.^26-29^ A variety of other factors may contribute to vulnerability or susceptibility to developing OUD, including genetics; genes such as OPRM1 and CN1H3 have been implicated.^30^ The Opioid Risk Tool questionnaire is a high sensitivity and specificity tool that can be used to identify patients at risk of aberrant behaviors who are prescribed opioids for chronic pain.^31^

Individuals with OUD are at increased risk of suicide attempts and suicide.^19^ However, medical conditions causing pain are also associated with increased suicide risk, and opioids may be implicated in intentional overdose in such circumstances.^32^ OUD frequently extends beyond the realm of health care in the hospital or clinical setting; individuals with OUD who are engaged in distribution and use of illicit substances may have limited interaction with health care systems but may instead interact with the criminal justice system.^33^

The present opioid epidemic in the United States is a complex problem driven by an amalgamation of factors. Misunderstanding the pathophysiologic underpinnings of pain, irresponsible prescribing practices, availability of diverted pharmaceutical products, and prevalence of illicit manufacturing have all contributed to the present public health crisis.^34^

## OPIOID CRISES IN THE UNITED STATES

The present opioid epidemic—considered the third major opioid crisis in the United States^35^—has historic antecedents.

### 1900s – First Opioid Crisis and Resulting Legislative Action

In the early 20th century, growing recognition of the prevalence of opioid misuse led to the International Opium Convention of 1912 that established a requirement for countries to domestically control and regulate substances such as opiates and cocaine.^36^ To address this requirement, the US Congress passed the 1914 Harrison Narcotics Tax Act. In addition to other regulations, the Harrison Act required medical professionals prescribing opium or opium-derived products to report the prescription to the Treasury Department on federally mandated forms, setting up a scenario of “the most comprehensive general criminal enforcement of any law against medical professionals in U.S. history”^37^ because according to the Treasury Department, maintaining chronic opioid use for the treatment of addiction was not legitimate medical practice. Consequently, Treasury agents prosecuted physicians and closed addiction treatment clinics.^38^ With the passage of the Harrison Act, “legal opioids became the exclusive province of physicians and pharmacists,”^39^ but physicians were dissuaded from prescribing opiates despite legitimate pain needs, and unexplained pain was frequently attributed to delusion, malingering, or outright abuse.^40^

### 1960s and 1970s – Second Opioid Crisis and Methadone Treatment

The second US opioid crisis is generally regarded as beginning in the 1960s and 1970s because of considerable heroin use. In response, a formal War on Drugs was initiated by the Nixon administration in 1971.^35^ US soldiers serving in Vietnam had high rates of heroin use and symptoms of dependence, although few became readdicted to heroin upon their return to the United States.^41^

The advent of methadone can be traced to the 1960s, a period in which OUD transitioned from a “disease of the mind, due to criminal or deviant behavior and a weak personality” to a “metabolic disease of the brain” requiring pharmacologic intervention.^42^ Methadone was found to be a keystone of a prolonged withdrawal program and its use was compared to using insulin to treat diabetic patients.^42^ Studies performed at Rockefeller University by Dole and Nyswander and by Kreek and Vocci showed that moderate to high doses (ie, 80 mg to 120 mg) of methadone dispensed in an outpatient clinical setting reduced or even entirely eliminated illicit opiate use, resulting in substantial reductions in morbidity and mortality.^43,44^ Methadone also reduced intravenous drug users’ injection frequency, a benefit that would later prove essential in reducing human immunodeficiency virus (HIV) transmission rates.^45^ In the 1970s, methadone maintenance therapy programs received federal support from the Nixon White House, leading to the rapid emergence of methadone maintenance clinics; however, methadone treatment was not established within the broader context of rehabilitation and other services.^44,46^ In reaction to this rapid emergence, strong counter-regulation measures were enacted by the US Food and Drug Administration (FDA), National Institute on Drug Abuse, and DEA via the Comprehensive Drug Abuse and Prevention Act, including limitations on admission criteria and duration of treatment, establishment of dose limits, and increased control of take-home doses.^44,46^ The passage of the Narcotic Addict Treatment Act in 1974 required annual registration of physicians and treatment centers.^44^ As a result, methadone clinics became limited in number. A 2022 study estimated that 18.2% of the US population does not have geographic access to a methadone clinic, with approximately 77,973 individuals in these areas likely to attend a clinic if geographic access barriers were removed.^47^

Despite the efficacy of methadone, several constraints limit its use. Because methadone is an opioid agonist, discontinuation is associated with withdrawal symptoms.^48^ Methadone increases the risk of arrythmias such as QT interval prolongation and torsades de pointes^49^ and is also associated with detrimental oral health effects.^50^ Many patients with OUD have comorbid conditions such as hepatitis, HIV, acquired immunodeficiency syndrome, and depression, and polydrug use is common; methadone may have drug-drug interactions with agents such as protease inhibitors, antimicrobials, anticonvulsants, calcium channel blockers, and selective serotonin reuptake inhibitors.^51^ As already stated, lack of access to methadone clinics is a key issue. Methadone use is also limited by factors such as variable attrition rates contingent upon dosage, the need for strong behavioral and psychosocial support, and its susceptibility to abuse and overdose.^42^ Patients may also experience issues with stigma secondary to methadone's use as a treatment for OUD.^46,52^

### 1980s and 1990s – Pain as the Fifth Vital Sign Campaign

In the 1980s and 1990s, undertreated pain became a focus of the medical community.^40^ Questions arose about why opioids were reserved for cancer pain and not used for other chronic pain conditions. The undertreatment of pain was criticized, and the interactions between providers and patients in the hospital, quality assurance standards, and drug regulations of the time were regarded as being unsupportive of pain recognition and treatment.^53^ Opioid prescribing increased in response to the “pain as the fifth vital sign” campaign.^18^ The Joint Commission and pain management specialists supported this initiative, to the extent that The Joint Commission developed standards recommending quantitative measurement of pain and emphasizing pain reduction.^54^ This shift gradually led to opioids becoming the primary mode of chronic noncancer pain treatment.^55^ Concurrently, in the late 1990s, opioid pharmaceutical manufacturers spread the misleading message that patients with severe or chronic pain would not become addicted to prescription opioids.^38^ Patients’ motivations for continued use of prescription opioids included coping with life stressors, self-medication for psychological and emotional issues, and the need for opioid maintenance to avoid withdrawal symptoms, with some patients even transitioning to heroin.^56^ Steadily, an opioid epidemic and public health crisis emerged.

### 1990s to the Present – Waves of the Third Opioid Crisis

The present opioid crisis can be defined temporally as occurring in a series of waves (Table 1).^5,57-60^ During the first wave, beginning in the 1990s, Purdue Pharma aggressively marketed a sustained-release oxycodone formulation (OxyContin) by providing all-expenses-paid symposia, using sophisticated marketing data to influence physician prescribing, and offering a lucrative bonus system to sales representatives; meanwhile, the company systematically misrepresented the risk of addiction.^61^ By 2004, a study of prescription drug abuse ranked the abuse of OxyContin and hydrocodone as the most prevalent and widespread.^62^

**Table 1.** Opioid Crisis Waves^5,57-60^

Wave	Year	Deaths per 100,000 Standard Population	Cause
First	1999	2.9	Increase in prescription opioid overdoses as a result of aggressive OxyContin (sustained-release oxycodone) marketing and prescribing
Second	2010	6.8	Increase in heroin overdoses
Third	2013	7.9	Increase in synthetic opioid overdoses, specifically from illicitly manufactured fentanyl
Fourth	2019	21.4	Increase in polysubstance use overdoses as a result of synthetic opioid and stimulant abuse during COVID-19

COVID-19, coronavirus disease 2019.

A second wave emerged beginning in approximately 2010, when heroin markets expanded to meet the demand of those addicted to prescription opioids.^57^ In 2013, a third wave began to form when highly potent synthetic opioids, specifically fentanyl, crowded the market.^5^ Despite reduced opioid prescribing (compared with 2010 to 2012, the prescribing rate declined 13.1% from 2012 to 2015), opioid deaths increased during 2013 to 2014 and 2014 to 2015 because of the rise of illicit drugs such as heroin and illegally manufactured fentanyl.^63-65^ Then overdose deaths involving all opioids, prescription opioids, and heroin decreased from 2017 to 2018: reductions of 2%, 13.5%, and 4.1%, respectively.^66^ These decreases were attributed to efforts to reduce prescriptions of high-dose opioids and the expansion of naloxone availability, as well as shifts from heroin to fentanyl, as deaths involving synthetic opioids increased 10% from 2017 to 2018.^66^

The COVID-19 pandemic was associated with significant increases in opioid-related overdose deaths in several US states.^67^ Additionally, a 10.1% increase in opioid overdose in the 12-month period between February 2019 and February 2020 was observed, primarily attributed to synthetic opioids, in conjunction with rising rates of stimulant abuse; this phenomenon is now regarded as the fourth wave of the present opioid crisis.^58^

Deaths from opioid overdose increased from 21,089 in 2010 to 47,600 in 2017, and then remained steady through 2019; in 2020, at the start of the COVID-19 pandemic, opioid-related deaths exceeded 68,000 (triple the deaths of 2010), and by 2021, more than 80,000 opioid overdose deaths were reported.^63^ In addition to deaths from overdose, significant morbidity and mortality from conditions such as sepsis and infective endocarditis have been observed in patients with OUD.^68,69^ Significant maternal and infant mortality in association with opioids has been noted as well.^70,71^

## LEGISLATIVE RESPONSE AND FDA APPROVAL OF BUPRENORPHINE

### 2000 – Drug Addiction Treatment Act and the X-Waiver

As the 1990s waned, growing awareness of the mounting opioid epidemic, combined with problems accessing needed treatment, led to a call for action. Access to care for OUD was limited by the few providers able to prescribe opioid substitution treatment. In an attempt to address the access problem, DATA 2000, passed by the 106th Congress on July 19, 2000, allowed practitioners to apply for a waiver to prescribe Schedule III, IV, and V opioid medications approved by the FDA for the treatment of opioid addiction.^72^ Under the act, the practitioner had to meet certain conditions: limit the total number of treated patients to 30 at one time, complete an 8-hour educational requirement, and obtain a second DEA number in addition to the standard DEA prescribing number.^72^ The second number began with an X, so it became known as the X-Waiver.^73^

### 2002 – Buprenorphine Approval and Office-Based Therapy

In 2002, the FDA approved 2 sublingual buprenorphine formulations to treat opioid addiction: buprenorphine (Subutex) and a combination tablet of buprenorphine plus naloxone in a 4:1 ratio (Suboxone).^74^ These medications were the only Schedule III, IV, or V medications that received FDA approval and were therefore eligible for use under DATA 2000.

Before DATA 2000 was enacted, the only opioid medications that could be used to treat opioid addiction were methadone and levo‐alpha‐acetyl‐methadol, and these medications could only be dispensed—not prescribed—in federally approved opioid treatment programs (ie, methadone clinics).^74^ DATA 2000 introduced the new paradigm of office-based opioid addiction therapy with buprenorphine.^75^

Buprenorphine/naloxone is considered first-line treatment for OUD relative to other medications such as full opioid agonists (eg, methadone), opioid antagonists (eg, naloxone, naltrexone), and alpha-2 adrenergic agonists (eg, clonidine, lofexidine).^76^ Advantages of buprenorphine/naloxone include similar if not greater efficacy, an extended duration of action, generally higher mu receptor affinity, favorable safety profile, and reduced diversion and misuse compared to methadone and clonidine or lofexidine (Table 2).^76-81^ A meta-analysis of 31 trials investigating maintenance rates of buprenorphine compared with placebo or methadone for OUD showed buprenorphine effectively maintained individuals with heroin dependence in treatment and suppressed illicit opioid use, particularly at doses above 2 mg, but retained fewer patients than methadone under flexible or low doses. The investigators also found that methadone demonstrated superior retention rates and comparable efficacy in suppressing illicit opioid use, emphasizing its clinical relevance over fixed-dose comparisons.^78^ A retrospective study of health datasets found that buprenorphine/naloxone was associated with lower illicit abuse or accidental overdose-related mortality and all-cause mortality relative to methadone.^79^ Drawbacks to buprenorphine treatment include exacerbated withdrawal symptoms when not tapered carefully or when used in combination with alcohol or benzodiazepines.^77^

**Table 2.** Relative Efficacy and Relative Side Effect Profiles of Pharmacologic Treatment Options for Opioid Use Disorder^76-81^

Drug Class	Drug	Relative Efficacy	Relative Side Effect Profile	Abuse Potential	Respiratory Depression Risk	Arrhythmia Risk	Hypotension, Sedation Risk
Partial mu receptor agonist-kappa receptor antagonist	Buprenorphine-naloxone	+++	+	++	++	+	+
Full mu receptor agonist	Methadone	+++	++	+++	+++	++	+
Alpha-2 adrenergic agonist	Clonidine, Lofexidine	++	+++	+	+	+	+++

Note: + indicates relative magnitude on a scale from + to +++.

Although they are less effective than buprenorphine/naloxone and methadone for managing opioid withdrawal, alpha-2 adrenergic agonists such as clonidine and lofexidine are recommended for use in settings such as prisons with less access to opioid agonists.^77^

Worth noting is that although the incorporation of naloxone was intended to discourage intravenous buprenorphine abuse, actual deterrence has not been proven.^80^ Diversion remains an issue. In countries where buprenorphine is widely available, such as France, illicit use and misuse have been documented, and in Finland, buprenorphine is the most widely abused opioid.^9^ Overall, however, the efficacy and safety profile of buprenorphine/naloxone makes it an advantageous first-line treatment for OUD.

### 2000 to 2022 – Ongoing Inaccessibility of Treatment with Buprenorphine

Despite the introduction of the X-Waiver in 2000, treatment of OUD with medications remained substandard during the ensuing 2 decades, primarily because of a lack of providers with X-Waivers. An analysis of the July 2012 DEA DATA Waived Physician List showed that only 16% of psychiatrists had an X-Waiver, the waivered physicians primarily practiced in urban areas, and nearly 30 million people in the United States (9.7% of the population) lived in counties without access to buprenorphine treatment.^82^ An electronic survey of 4,225 US clinicians conducted between March and April 2018, revealed that only 13.1% of providers with X-Waivers had prescribed at or near their patient limit during the prior month, and many were not prescribing at all.^83^ In 2019, only 102,570 US clinicians were waivered, and many of them were not treating patients with OUD.^84^ Specialty-specific figures also reflect this exiguity. In a 2020 study of 31,211 obstetrician-gynecologists, only 560 (<2%) had obtained an X-Waiver, despite significantly increasing national rates of neonatal abstinence syndrome in infants born to women with OUD.^85^ A 2022 mixed-methods survey study conducted by Lanham et al showed that only 61 (48.4%) of 126 clinicians—most of whom were working in primary care, psychiatry, or general acute care settings—had received an X-Waiver,^86^ and among the providers with an X-Waiver, only 36% were prescribing buprenorphine. The surveyed clinicians cited the following barriers: complexity of the X-Waiver process, lack of professional support and referral network, getting started, and obtaining reimbursement for treatment.^86^ Additional barriers to obtaining an X-Waiver reported by Russell et al were lack of training and mentors, fear of a DEA audit, and lack of time and money to integrate the services into busy primary care offices.^87^ In a Kentucky study, physicians without an X-Waiver were less likely to report positive personal beliefs about using medications to treat OUD compared to physicians who had an X-Waiver.^88^ While the recommendation to include X-Waiver requirements as a part of residency training were proposed in the literature,^89^ implementation remained limited.

### 2006 to 2023 – Evolution of the X-Waiver

Several changes were made to the X-Waiver requirements with the goal of increasing buprenorphine accessibility. In 2006, an amendment to the Controlled Substances Act increased the patient cap from 30 patients during the first year to 100 patients thereafter.^90^ In 2016, the Comprehensive Addiction and Recovery Act expanded the categories of practitioners who could prescribe medications for OUD to include nurse practitioners and physician assistants after they completed 24 hours of training.^91^ In August 2016, a final rule from the Substance Abuse and Mental Health Services Administration increased the maximum number of patients that a practitioner could treat for OUD to 275.^91^

In April 2021, the US Department of Health and Human Services exempted eligible providers treating ≤30 patients from the X-Waiver educational requirement of 8 hours of training; however, providers treating >30 patients with buprenorphine were still required to complete the training.^92^ One reason for this change in policy was to increase the ability of emergency medicine physicians to prescribe buprenorphine.^93^

However, recognition that the X-Waiver was a fundamental impediment to buprenorphine accessibility continued to grow, and data from a grant-funded program designed to increase the number of waivered providers indicated that removing the training requirement alone was not likely to result in major changes to prescription rates.^87^

With the signing of the Consolidated Appropriations Act of 2023, also known as the Omnibus Bill, the X-Waiver was entirely eliminated, patient limits were removed, and providers only needed a DEA registration number to prescribe buprenorphine.^94,95^ Separate from the repeal of the X-Waiver, however, the Omnibus Bill introduced new training requirements for new or renewing DEA registrants: a total of 8 hours of opioid or other substance use disorder training; or board certification in addiction medicine or addiction psychiatry; or graduation within 5 years and good standing status from medical, advanced practice nursing, or physician assistant school that included at least 8 hours of an opioid or other substance use disorder curriculum.^94^

## NEXT STEPS

The repeal of the X-Waiver could prove to be a crucial step in increasing the availability of and access to medications to treat OUD. However, X-Waiver repeal is far from the last step in the crusade against the opioid epidemic. Understanding the causes of OUD, widespread acceptance of the efficacy and utility of medications used to treat OUD, recognition of addiction treatment as a core competency of the generalist, and a coordinated approach to combat disparities and stigma will all be needed to make significant gains in quelling the crisis.

Addressing the precipitating causes of the increase in OUD rates, such as opioid prescribing and patient mindsets, is particularly important. Chronic pain requires effective treatment, and a variety of effective nonopioid analgesics are available, including nonsteroidal anti-inflammatory drugs, antidepressants, anticonvulsants, skeletal muscle relaxants, topical analgesics, and inflammatory mediators.^96^ For some patients, buprenorphine may be an effective agent for the management of chronic pain, with considerable safety advantages compared to full opioid receptor agonists.^97^ As the understanding of the pathophysiologic underpinnings of chronic pain evolves, providers must take care to avoid the undertreatment of chronic pain, which can lead to psychiatric comorbidities such as anxiety, depression, or suicidality.^98,99^ In patients with physiologic and psychologic stress, cognitive processing errors such as catastrophizing have been associated with greater risk of prescription opioid misuse; concerted efforts by providers to assess and mitigate such thought processes may lead to decreased likelihood of OUD.^100^

Changes can also be made to mitigate the risks and adverse effects of medications used to treat OUD. For example, the novel approach of buprenorphine microdosing may alleviate some of the opioid withdrawal symptoms associated with the typical initiation of the medication.^101^ Extended-release buprenorphine formulations in the form of long-acting monthly injectables have shown efficacy for OUD treatment.^102^ Long-acting buprenorphine/naloxone injectables offer additional benefits compared to traditional oral formulations, such as greater convenience, greater adherence, reduced treatment cost, and little to no withdrawal symptoms upon cessation; moreover, significant reductions in the risks of diversion, nonmedical use, takeaway treatment doses, and stigma have been reported.^103^ However, patients using long-acting injectables still require psychosocial support interventions such as addiction counseling, peer support, and contingency management to ensure effective treatment for OUD.^104^

Beyond technical improvements to treatment, other possibilities for improving OUD treatment exist. Russell et al, for instance, suggested that initiatives must include an effort to normalize prescribing buprenorphine in primary care settings through direct exposure of practitioners to patients receiving medications for OUD, instruction beginning in undergraduate medical education to decrease stigma, and the establishment of trust around disclosure with patients in a primary care setting.^87^ Because patients with OUD may also have conditions such as depression and suicidality, providers must also emphasize treating these comorbidities to prevent relapse and reduce the risk of overdose fatality.^21^ Increased institutional and leadership support have also been identified as an integral aspect of changing attitudes toward buprenorphine prescribing.^105,106^ In a 2022 qualitative analysis of 22 semistructured interviews with hospitalists in Philadelphia, Pennsylvania, the X-Waiver was cited as only 1 of several barriers to buprenorphine prescribing; other barriers the hospitalists identified were the lack of training in and experience with OUD, lack of community OUD treatment infrastructure, and lack of inpatient OUD/withdrawal treatment resources.^107^ Increased availability of training and educational materials for medications used to treat OUD in general and buprenorphine specifically could potentially help to increase buprenorphine prescribing.

Racial disparities in the care of OUD patients are also pervasive. A 2022 study of a 20% random sample of nonprofit hospitals in the United States found that the availability of common services such as programs to increase access to addiction treatment services, prescriber guidelines, and targeted risk education and harm reduction were substantially lower in hospitals serving communities with high percentages of Black or Hispanic residents.^108^ Lagisetty et al conducted a retrospective study to determine buprenorphine prescription rates by race/ethnicity in 205,245 outpatient visits occurring from 2012 to 2015 and found that the odds for non-White patients to obtain buprenorphine prescriptions were significantly lower than for White patients.^109^ In a 2023 secondary analysis of 21 emergency departments across 5 health care systems, despite adjusting for clinician X-Waiver status and other factors, Black patients were less likely to receive buprenorphine than White patients.^110^ Hence, even in a post-X-Waiver landscape, patients of diverse backgrounds continue to face limitations to buprenorphine access.

Analogous to the way conditions such as diabetes and hypertension are treated with lifestyle changes and medication, OUD also calls for a multifaceted approach. Long-standing preconceived notions that are stigmatizing—such as assumptions that all patients with OUD are disruptive to hospital settings, manipulative, or drug seeking—may be counterproductive to effective OUD treatment.^111,112^ Instead, the best care for these patients may be achieved with empathy and understanding, and such care may be encouraged through early influential encounters with OUD patients during medical training.^113^ An emphasis on the overall psychosocial well-being of the patient, rather than the simple absence of symptoms, promotes comprehensive treatment of OUD.

## CONCLUSION

The public health crisis of OUD has had a significant impact in the United States. The repeal of the X-Waiver may be an important step in increasing the availability of buprenorphine, a primary treatment modality for OUD. However, substantial work remains to reduce stigma, address psychiatric comorbidities, address racial disparities, and show empathy when treating patients with OUD. The most efficacious treatment may occur when OUD therapy is integrated with treatment for the patient's other medical and psychological problems and by taking the patient's psychosocial well-being into account. Institutional support with cooperation among providers and leadership may also play a key role in forming the basis of an effective OUD treatment program. Ultimately, improved access to safe and effective treatment options may help to manage the opioid public health crisis.

## Notes

*This article meets the Accreditation Council for Graduate Medical Education and the American Board of Medical Specialties Maintenance of Certification competencies for Patient Care and Medical Knowledge.*
